# Biomimetic Hybrid Systems for Tissue Engineering

**DOI:** 10.3390/biomimetics5040049

**Published:** 2020-10-09

**Authors:** Omid Yousefzade, Ramaz Katsarava, Jordi Puiggalí

**Affiliations:** 1 Departament d’Enginyeria Química, Universitat Politècnica de Catalunya, Escola d’Enginyeria de Barcelona Est-EEBE, 08019 Barcelona, Spain; o.yousefzade@gmail.com; 2 Institute of Chemistry and Molecular Engineering, Agricultural University of Georgia, Kakha Bedukidze Univesity Campus, Tbilisi 0131, Georgia; r.katsarava@agruni.edu.ge

**Keywords:** tissue engineering, biomimetic proteins, biomimetic DNA, biomimetic scaffolds, melt electrospinning, hybrid technologies, hard and soft tissues regeneration, stem cells

## Abstract

Tissue engineering approaches appear nowadays highly promising for the regeneration of injured/diseased tissues. Biomimetic scaffolds are continuously been developed to act as structural support for cell growth and proliferation as well as for the delivery of cells able to be differentiated, and also of bioactive molecules like growth factors and even signaling cues. The current research concerns materials employed to develop biological scaffolds with improved features as well as complex preparation techniques. In this work, hybrid systems based on natural polymers are discussed and the efforts focused to provide new polymers able to mimic proteins and DNA are extensively explained. Progress on the scaffold fabrication technique is mentioned, those processes based on solution and melt electrospinning or even on their combination being mainly discussed. Selection of the appropriate hybrid technology becomes vital to get optimal architecture to reasonably accomplish the final applications. Representative examples of the recent possibilities on tissue regeneration are finally given.

## 1. Introduction

Nowadays, great efforts are focused on the design of biodegradable scaffolds for applications in the biomedical field such as tissue engineering and organ regeneration. Natural and synthetic polymers and even their blends have been tested, but an ideal system has not yet been developed due to the multiple requirements that must be met. These involve chemical, physical, and biological properties. For example, the rate of degradation must be adjustable and the corresponding degradation products must not be toxic, the scaffolds produced from the selected material must retain their mechanical integrity before regeneration, the material should guarantee immunogenicity, and even should have capacity to tune cell adhesion, proliferation, and differentiation. Scaffolds with multifunctional properties (e.g., mechanical performance, biocompatibility, surface adhesion) are consequently often required, being the best options focused on the use of hybrid systems that combine different materials [1,2].

Incorporation of stem cells in the scaffolds appears fundamental to enhance healing processes. These cells can be differentiated under the action of an opportune stimuli while homeostasis in the healthy tissue could be kept. Performance of designed scaffolds can be improved by the additional incorporation of active agents to stimulate tissue regeneration (e.g., growth factors) and even to avoid infection risk and biofilm formation (e.g., bactericide drugs) [3,4].

Usually, scaffolds developed for tissue engineering try to mimic the natural extracellular matrix (ECM). This is constituted by different entrapped proteins (e.g., collagen, laminin, or fibronectin) that act as cell binding ligands. Specifically, integrin-recognizing peptide sequences are fundamental since they provide an inherent cell adhesivity [1,5] between the cellular cytoskeletons and the ECM microenvironment.

A good control of porosity, interconnectivity between pores, and even the size of pores is fundamental to provide appropriate 3D-scaffolds for the different kinds of tissues (e.g., hard or soft). Therefore, great efforts are focused to the development of new processing technologies able to achieve the indicated goals. In fact, macro-, micro-, and nanoarchitecture of 3D scaffolds have a primordial relevance to replicate the structural complexity of living tissues. Hybrid systems considering both material mixtures and a combination of fabrication processes are nowadays fundamental to mimic natural tissues by providing multiphasic or multimaterial structures [6,7,8].

The present review is structured in four well differentiated parts that are focused on (a) materials based on natural polymers that are employed for tissue engineering applications; (b) development of synthetic polymers able to mimic natural proteins and DNA; (c) technologies for preparation of optimal scaffold architectures; (d) specific applications of biomimetic scaffolds on tissue regeneration.

## 2. Biomimetic Hybrid Systems Based on Natural Polymers for Tissue Engineering

Tissue engineering applications require the use of complex scaffolds where an accurate selection of materials is essential. In addition, it is advantageous that these materials could accommodate growth factors and cells, while providing cues to guide cell adhesion and proliferation [9,10].

**Figure 1:**
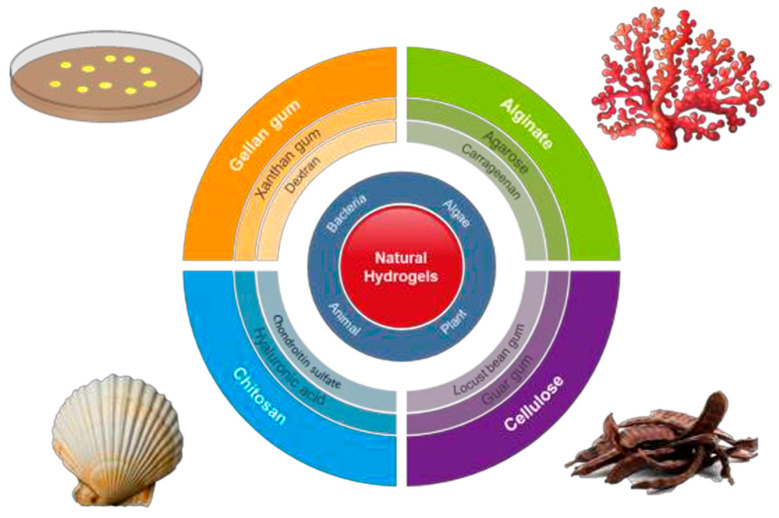
Natural polysaccharides able to produce hydrogels and respective sources. Reproduced with permission from [9].

Hydrogel scaffolds appear ideal matrices to culture cells and produce in vitro tissues [11]. Probably, polysaccharides (Figure 1) constitute the most interesting family of polymers due to their high hydrophilicity, their origin linked to the living tissues, their biodegradable and biocompatible properties, their ability to render hydrogels with similar properties to ECM [12], and their capacity to display bio-responsive functions [13]. Nevertheless, chemical modifications appear necessary to facilitate the attachment of cells, which may be a problematic feature since a complete removal of employed toxic agents is essential for subsequent biomedical applications [14]. Direct blending of polysaccharides is a recent strategy that is applied to improve cellular adhesion and proliferation as recently reviewed by Ng et al. [9].

Cellulose and chitosan are between the most employed hydrogels for biomedical applications. In fact, they are the most abundant natural polymers in earth. Cellulose appears an ideal matrix for tissue engineering applications [15], alone or even blended [16] with other polymers like chitosan. Hydrogels can be prepared by crosslinking of aqueous cellulose esters [17], a procedure that avoids the limitations caused by insolubility of natural cellulose in aqueous media. Nanocellulose (i.e., cellulose nanocrystals [18], CNCs, cellulose nanofibers [19], NFCs, and bacterial cellulose [20], BC) can also be incorporated into polymer matrices to improve mechanical properties. For example, the interpenetrating network of gelatine and alginate reinforced with CNCs can improve the performance of natural cartilage, favor cell adhesion, and protect cells from immune rejection [21]. A second interesting example about the use of nanocellulose corresponds to the development of BC membranes coated with alginate and collagen in each side [22].

Deacetylation of chitin leads to chitosan, which has a great structural stability, capability to absorb water, and a cationic character that favors the formation of gel particles through electrostatic interactions [23]. Chitosan is mostly employed as injectable hydrogels, crosslinking agents like genipin [24] and even hydroxyapatite particles being added to improve osteogenic properties and enhance cell adhesion and proliferation [25].

Collagen is the most relevant structural protein in the human body [26] and is the major component of the native ECM. Therefore, great efforts have been focused on preparing collagen-based scaffolds to mimic native environment [27]. Solution processing (e.g., electrospinning) of collagen has some restrictions since solvents used to denaturalize the highly insoluble triple helix structure (e.g., 1,1,1,3,3,3-hexafluoroisopropanol) should be avoided due to their toxicity [28]. To this end, co-electrospinning has been proposed using a biodegradable synthetic polymer able to provide elasticity (e.g., the copolymer of l-lactide and ε-caprolactone) and a mixture of collagen and a water-soluble sacrificing polymer (e.g., PVP) that acts as a transporter polymer for an easy spinning of collagen [29].

Different scaffolds have successfully been developed to mimic the 3D organization of interstitial ECM, but scarce results have been reported concerning the reproducibility of the 2D ECM basement membrane (BM) [30,31]. These membranes establish functional polarization of epithelial and endothelial cell layers throughout the entire body [32,33] and appear fundamental for artificial organ technologies [30,34]. Furthermore, BMs assure tissue compartmentalization and may prevent the spreading of cancer cells [35]. Basically, BMs are based on the assembly of two different proteins: type IV collagen (Col IV) and laminin (LM), which provides unique mechanical properties for an effective protection of tissues from external stresses [36]. Artificial matrices mimicking BMs have been prepared by co-assembling polylaminine and Col CV under acidic conditions. A layered morphology (thickness around 15 m) was derived, its great capacity to support keratinocytes and form a cell layer close to the architecture of natural epidermis being demonstrated [31].

Gelatine, as a soluble denaturalized collagen, is widely employed for tissue regeneration due its relevant role in cellular metabolism and morphogenesis. Moreover, gelatine is interesting for giving rise to thermosensitive scaffolds taking into advantage its capability to form solid gels at low temperature through intermolecular hydrogen bonding interactions [37], which can be disturbed at increasing temperatures to form liquid gels.

Mixtures of gelatine with other natural polymers like agar and alginate have also been evaluated. Thus, agar–gelatine mixtures have been found appropriate to produce hydrogel scaffolds with a uniform internal pore structure [38], and gelatine/alginate mixtures were found suitable for manufacturing biological scaffolds by 3D bioprinting [39]. Natural gelatine has also been blended with synthetic polymers such as nylon 6 and polyurethane to render suitable scaffolds for bone tissue engineering [40]. New scaffolds facilitated apatite-like mineral deposition, and promoted osteoblast cell attachment, migration, and proliferation [41]. Hybrid nanofibrous scaffolds have also been prepared by layering a poly(3HB-*co*-4HB) copolymer and gelatine [42]. The trilayered system with gelatine in the middle showed a good water-resistant ability and high proliferative activity of mouse fibroblasts. Hydrogels based on gelatine and methacrylamide have been found appropriate to simulate liver tissue, a hepatocyte cell survival rate of 97% being found [43]. It can be deduced that hybrid composite materials with natural and synthetic polymers are gaining relevance to develop biomimetic scaffolds [44,45,46].

Fibrin, keratin, alginate, and hyaluronic acid are other relevant natural polymers that are gaining relevance for the development of biomimetic scaffolds. Fibrin is a structural protein employed to prepare bioprinted scaffolds [47]. Specifically, scaffolds with embedded neurons have been, for example, prepared by a layer-by-layer printing process These materials can attain modulus and tensile strength of 2.9 and 1.7 MPa, respectively [48]. The fast gelation rate of fibrin can be combined also with the high biocompatibility of collagen to render scaffolds with fast wound closure and high vascularization [49].

Keratin is a structural fibrous protein that forms part of epidermal structures (e.g., nails, wool, hair, and feathers) and provides toughness and resilience depending on the number of sulfur cross-links that are established through their cysteine units [50,51]. Keratin can be extracted from natural materials by different processes such as the use of ionic liquids [52], microwave irradiation [53], alkaline treatment [54], and reduction of disulphide linkage [55]. Capability of keratin to self-assemble and support cellular proliferation enhance its use in tissue regeneration applications [50,56]. Between them, the reconstruction or regeneration of ocular surface [57], urinary track [58], and nerves [59] are significant. The relatively poor mechanical properties and brittle structure of keratin justified its blending with different synthetic (e.g., poly(ε-caprolactone (PCL), polylactide (PLA), polyvinyl alcohol, and polyethylene oxide) and natural (e.g., chitosan, gelatine, silk, fibrin, and hyaluronic acid) polymers [51]. 

Sodium alginate is a natural polysaccharide widely employed in tissue engineering despite some limitations like low mechanical integrity to form suitable fibers and too high biodegradation rate to be used as a supporting matrix. Nevertheless, this polysaccharide has several advantages for cartilage tissue engineering as recently reviewed [60].

Hyaluronic acid (HA) is a glycosaminoglycan that can give rise to fibrillar structures with good mechanical performance and a regulatory function [61]. HA has interest in regeneration processes [62] and consequently is considered in tissue engineering applications. Fabrication of HA scaffolds with high porosity (i.e., 70–95%) and swelling ratio (i.e., 2000–6000%) has been reported. These materials display soft-tissue mimetic mechanical properties (≈0.5–1.5 kPa), accelerated tissue formation, neovascularization, and reepithelialization in vivo [63]. 

**Figure 2:**
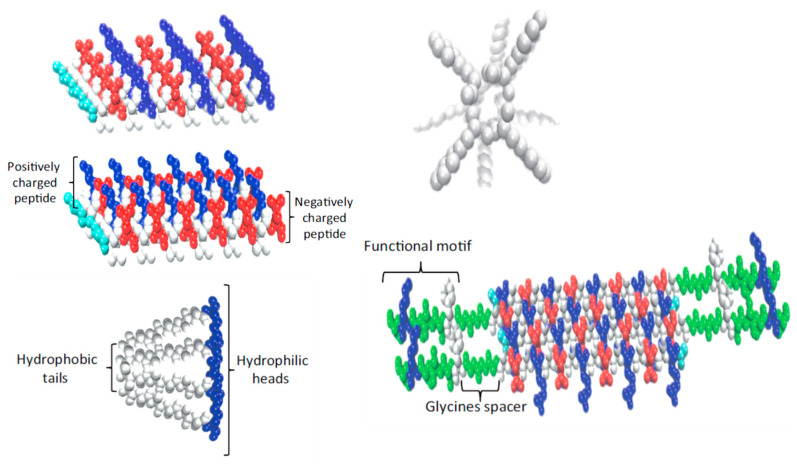
Strategies developed to favor peptide self-assembly: alternate disposition of hydrophilic and hydrophobic residues, complementary co-assembling peptides, peptide amphiphiles, cyclic peptides, and functionalized peptides. Reproduced with permission from [70].

Interest towards peptide hydrogels is emerging despite their higher cost with respect to typical polysaccharide-based materials. Efforts are logically focused to mimic the natural fibrillar proteins of ECM by means of synthetic peptides able to self-assemble. Different morphologies are derived depending on the peptide sequence. Basically, strategies to favor self-assembly are based on (a) an alternate disposition of hydrophilic and hydrophobic residues (e.g., Arg-Ala-Asp-Ala characteristic of the named RADA peptides) [64]; (b) the use of complementary peptides (i.e., peptides having opposite electric charges) [65]; (c) peptide amphiphiles [66]; (d) cyclic peptides [67]; (e) functionalized peptides [68] (Figure 2). High cost, non-renewability, complex purification processes, and demanding storage conditions are some drawbacks that limited the use of protein-based hydrogels despite their inherent cell adhesivity [69,70].

Electrostimulation (ES) has beneficial effects on tissue regeneration (i.e., muscle, bone, skin, nerve, tendons, and ligaments) since it enhances cell proliferation, ECM synthesis, cytokines production, and vasculature development [71]. The development of scaffolds based on electroactive polymers is highly interesting to facilitate the successful application of ES. Conductive polymers (i.e., based on electronic conductivity enabled through oxidized pi-bond conjugation states) are interesting but have poor properties (in general are brittle and non-degradable thermosets with a conductivity that decreases progressively as a reduced state is attained). Usually, composite systems based on conductive polymers like polypyrrole, polyaniline, and polythiophene derivatives, and natural polymers like chitosan, cellulose, and alginate have been selected. Alternatively, the incorporation of ionic functional groups (e.g., carboxylic, sulfonic, and amine) to a polymer can generate ionic conductive materials without modifying significantly biocompatibility and degradation profiles [72]. 

Incorporation of carbon nanotubes promote cell proliferation and facilitate nerve regeneration. Conductive hydrogels have been prepared from *N*-isopropylacrylamide (NIPAM), laponite, and CNTs [73], also being remarkable the derived improvement of mechanical properties. This characteristic was considered to prepare hybrid hydrogels constituted by gelatine, sodium alginate, and CNTs to be employed as bioink to print hollow tubular scaffolds. These led to blood vessels after colonization by epidermal fibroblasts. Doping with CNTs was essential to enhance mechanical properties while no cytotoxicity was found [39]. 

## 3. Synthetic Polymers Able to Mimic Proteins and DNA

### 3.1. Biomimetics of Proteins—Artificial Polymers Made of α-Amino Acids

As discussed above proteins are widely applicable materials for numerous biomedical applications—in tissue engineering, drug delivery systems, treatment of infectious diseases, etc. Furthermore, they can provide structural support in their fiber form, and properties such as heat shock, self-assembly, and bioactivity. Proteins represent an important family of naturally occurring polymers having a unique property to “disappear” from the body after fulfilling assigned functions. After biodegradation the proteins release natural endogenous products—α-amino acids (AAs) that are nutrients for cells. Another attractive feature of the proteins is a high affinity with tissues (tissue compatibility) owing to hydrophilic amide (peptide) NH-CO bonds.

However, in spite of a great practical potential of proteins as biomaterials they have some substantial limitations among which the most important are immune rejection along with risk of disease transmission and batch-to-batch variation [74]. The undesirable immune reaction of proteins is caused by the proteinaceous molecular architecture of collagen (proteins in whole). It is known that α-amino acids (AAs) in protein molecules (backbones) have so-called “head-to-tail” orientation (assuming that α-amino group is “head” and α-carboxyl groups are “tail”) [75,76,77,78]. Besides, proteins contain only amide NH-CO (peptide) groups in the backbones that significantly restricts their material properties. For these reasons, the synthetic AAs-based polymers (AABPs) having a non-proteinaceous molecular architecture seem [75,76,77,78] more promising for biomedical applications. Such polymers can contain, along with NH-CO bonds, additional hetero-bonds such as ester, urea, urethane, etc., in the backbones—thereby increasing the range of material properties and providing low to zero immunogenicity of the polymers. 

We shall now discuss the main families of artificial AABPs—biomimetics of naturally occurring polymers—proteins.

#### 3.1.1. Poly(α-Amino Acid)s, PAAs

**Figure 3:**
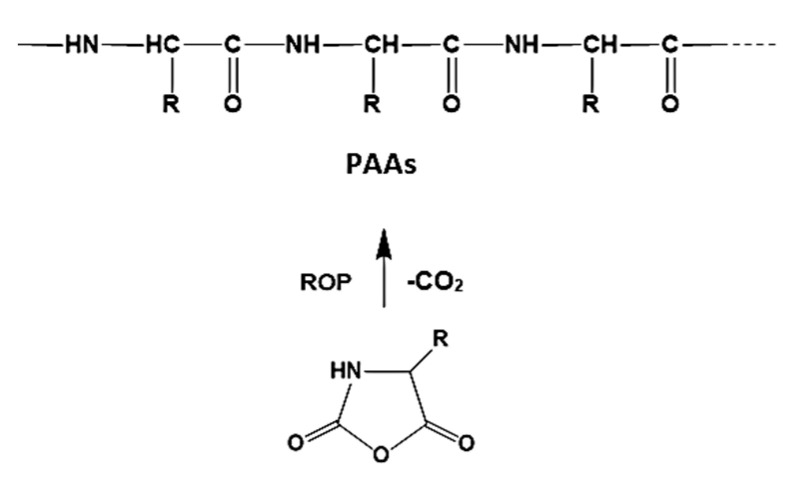
The synthesis of poly(α-amino acid)s (PAAs) via ring-opening polymerization (ROP) of *N*-carboxy-α-amino acid anhydrides (NCAs).

Owing to the presence at least two functional groups (α-amino and α-carboxyl), the AAs can be used as monomers for creating synthetic analogues of proteins—poly(α-amino acid)s (PAAs). The PAAs were the first artificial polymers made of AAs. They have conventional proteinaceous “head-to-tail” molecular architecture and were synthesized and studied by many famous chemists [79,80,81,82]. The preparative methods developed make possible the synthesis of a large variety of PAAs in a wide range of average molecular weights. A number of different derivatives of AAs and peptides have been used as monomers for the preparation of PAAs via AB-type step-growth polymerization (solution polycondensation of hetero-bifunctional monomers) or by Merrifield solid phase peptide synthesis [81]. However, while this strategy comprises multistage synthesis of di, tri, or higher peptides, it is complex and expensive. More rational and, hence, commonly used way of the synthesis of PAAs is ring-opening polymerization (ROP) of appropriate cyclic monomers—*N*-carboxy-α-amino acid anhydrides (NCAs). As it is shown in Figure 3 the ROP proceeds with evolution of carbon dioxide resulting in high-molecular weight PAAs [81].

The PAAs could also be synthesized via thermal polycondensation of AAs derivative as it was done for *trans*-4-hydroxy-l-proline methyl ester. The thermal polycondensation resulted in functional poly(*trans-*4-hydroxy-l-proline with Mw ≤ 3600 Da [83].

The PAAs were explored as potential new biomaterials starting from about 1970 [80]. The studies revealed that most of the PAAs are less suitable biomedical materials due to their immunogenicity owing to the proteinaceous molecular architecture [81]. Besides, PAAs showed unfavorable physical-chemical characteristics—with few exceptions, PAAs tend to be insoluble polymers that decompose in the molten state [79]. Most synthetic PAAs therefore cannot be processed into shaped objects by conventional fabrication techniques. Synthetic PAAs also tend to be expensive, partly because of the need to secure rather unstable *N*-carboxyanhydrides as the monomeric starting materials. All these factors limit the range of practical applications for synthetic PAAs [82,83]. So far, only a small number of poly(γ-substituted glutamates) and copolymers thereof are considered as promising candidates for biomedical applications [84]. Among applicable PAAs are also poly(α-lysine) and polycysteine suitable as drug carriers, and cationic polymers such as polyarginine and polyhistidine which form polyplexes with nucleic acids and are suitable for gene delivery purposes [75]. Hydrophobic PAA such as poly(l-phenylalanine) and its copolymers having no active lateral functional groups were used for fabricating nanosized vesicles, micelles, or nanoparticles suitable as hydrophobic drug carriers [85].

#### 3.1.2. Pseudo-poly(Amino Acid)s

Pseudo-poly(amino acid)s (PPAAs) represent artificial AABPs in which α-l-amino acids or dipeptides are polymerized by various chemical bonds (ester, iminocarbonate, etc.) involving the functional groups located on the amino acid side chains, rather than the amino acid termini [86,87]. Accordingly, PPAAs have a non-proteinaceous molecular architecture [75,76,77,78] and differ from conventional PAAs which have a proteinaceous molecular architecture (see Section 3.1.1 above). This approach allows to construct artificial polymers from naturally occurring compounds such as AAs but free of shortcomings of conventional PAAs discussed above. PPAAs could be synthesized from suitably modified polyfunctional AAs via either (i) homopolymerization or (ii) copolymerization with non-AA monomers (diacids, diols, diamines, diisocyanates, etc.) through a variety of reactions, leading to PPAAs of various classes—polyesters, polyamides, polyureas, polyurethanes, polyarylates, polycarbonates, poly(iminocarbonate)s, etc., with a wide range of structures and properties [75,76,77]. The process (i) belongs to AB type step growth polymerization (SGP) or ring-opening polymerization (ROP), and process (ii) to AABB type SGP. Some of the synthetic approaches to and representatives of the PPAAs are discussed in brief below. 

**Figure 4:**
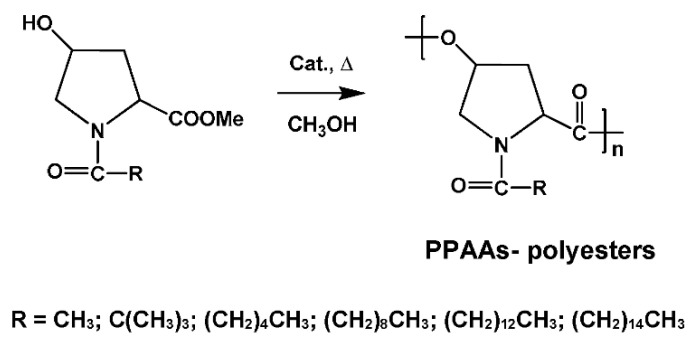
Synthesis of the pseudo-poly(amino acid)s (PPAAs)-polyesters (PEs) by thermal polycondensation of *N*-substituted *trans*-4-hydroxy-proline methyl ester.

One of the first representatives of PPAAs were polyesters (PEs) made from naturally occurring hydroxy-AAs such as hydroxyproline and serine [86,87,88,89]. The PPAA-PEs can be synthesized from modified (*N*-substituted) hydroxy α amino acids either by AB type SGP or by transforming them into corresponding β-lactones and subsequent ROP. In the case of *trans*-4-hydroxy-proline direct thermal polycondensation (AB type SGP) of suitably modified AA (Figure 4) led to high-molecular-weight PPAA-PEs [86,87].

**Figure 5:**
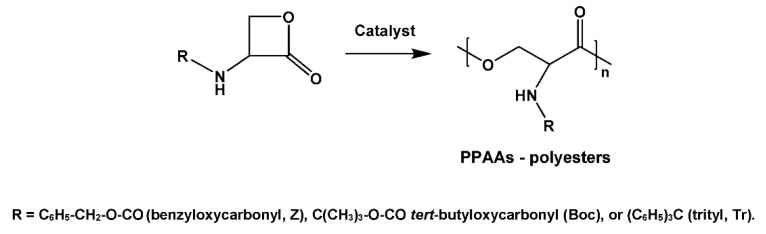
Synthesis of the PPAA-PEs by ROP of *N*-protected serine.

In the case of AA serine [88,90] high-molecular-weight PPAA-PEs were obtained by transforming them into corresponding β-lactones and subsequent ROP (Figure 5).

**Figure 6:**
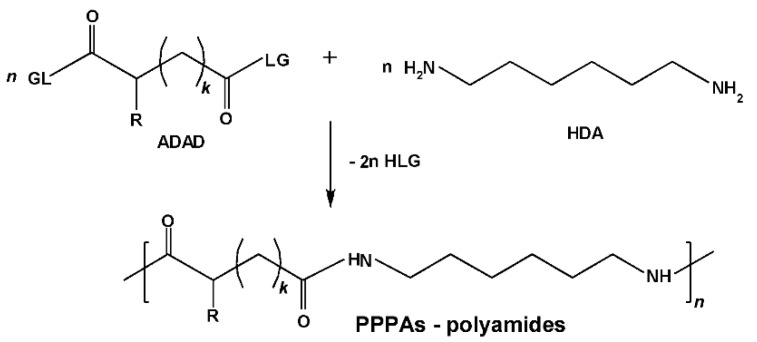
Synthesis of PPAA-polyamides (PAs) on the basis of *N*-protected α-amino dicarboxylic acids.

A variety of PPAAs were obtained by copolycondensation (AABB type SGP) of suitably modified polyfunctional AAs with non-AA monomers. High-molecular weight polyamides were synthesized by copolycondensation of activated diesters (ADAD) of trifunctional AAs—*N*-protected aspartic and glutamic acids with a fatty diamine (e.g., 1,6-hexanediamine (HAD) as depicted in Figure 6, for leaving groups (LG) and other details see [75,76,77] and references cited therein).

**Figure 7:**
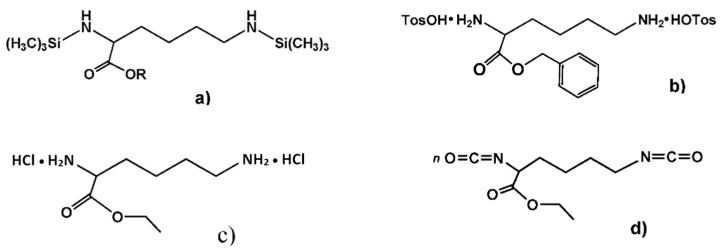
Monomeric forms of α-amino acid (AA) lysine; (**a**–**c**) lysine-based bis-nucleophiles, and (**d**) -lysine-based bis-electrophile.

A variety of suitable monomers—both bis-nucleophiles (Figure 7a–c) and electrophiles (Figure 7d)—were obtained on the basis of esters (i.e., C-protected) of trifunctional AA l-lysine and used for synthesizing high-molecular-weight PPAAs by polycondensation with different types of bifunctional counter-partners. As a result, a number of the PPAAs of various classes—polyamides, polyureas, polyurethanes, functional polymers, etc.—promising as drug carriers were obtained [75,76,77]. 

**Figure 8:**
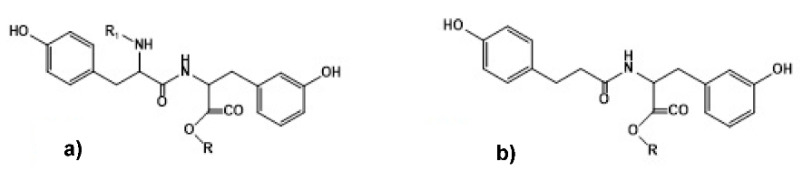
Chemical structure of “physiological” bis-phenols—(**a**) *C-* and *N-*protected tyrosine dipeptide and (**b**) C-protected desaminotyrosyl-tyrosine. R = C_2_H_5_, C_4_H_9_, C_6_H_13_, C_8_H_17_, C_12_H_25_.

One of the most promising representatives of PPAAs are the polymers made from naturally occurring α-amino acid tyrosine [86,91]. The PPAAs are synthesized via tyrosine-based “physiological” bis-phenols such as tyrosine dipeptide and desaminotyrosyl-tyrosine (Figure 8a,b) as key monomers (lateral phenolic groups of AA tyrosine are used for constructing the polymeric backbones). These monomers were used for synthesizing all classes of heterochain polymers available from bis-phenol type monomers and linked by non-amide bonds such as ester, ether, carbonate, or iminocarbonate, etc. (see Section 3.2 below).

The PPAAs show a wide range of material properties suitable for their applications as both resorbable drug delivery systems and surgical materials [75,76,77,86,91]. It was shown by Kohn et al. [92,93] that the PPAAs can be the promising polymers for implantable biodegradable orthopedic devices and in bone-tissue engineering due to the desired hydrolytic degradation and descriptive histologic evaluation.

#### 3.1.3. Polydepsipeptides

The next representatives of the synthetic AABPs are polydepsipeptides (PDPs)—an important class of biodegradable biomaterials composed entirely of naturally occurring (“physiological”) building blocks such as α-amino acids and α-hydroxy acids. Initially, AB type PDPs were synthesized by solution polycondensation of di-, tri-, or higher linear [94]. However, this multistep synthesis is complex and expensive and less suitable for large-scale preparation of high-molecular-weight PDPs. 

**Figure 9:**
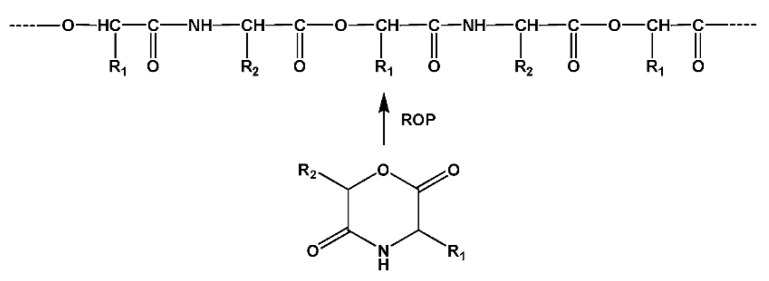
Synthesis of polydepsipeptides by ROP of cyclic depsipeptides.

A more rational method of the synthesis of AB type PDPs consists of ring-opening polymerization (ROP) of cyclic depsipeptides—morpholine-2,5-diones as depicted in Figure 9 [95]. 

Subsequently, by the ROP and copolymerization of various cyclic monomers—morpholine-2,5-diones—a huge variety of degradable polymers including random and alternating diblock and triblock, as well as graft PDPs were synthesized (see [75,76,77] and references cited therein). At this, PDPs could be obtained either free of or with lateral functional groups including hydroxyl, amine, thiol, or carboxyl functions depending on the structure of the AA used. Physical-chemical properties and degradation rates of the PDPs can be adjusted by varying α-hydroxy acids and AAs (i.e., by varying R_1_ and R_2_ substituents of 2,5-dioxomorpholine rings, Figure 9). It has to be noted that, though in the backbones of AB type PDPs the AAs are inserted in “head-to-tail” manner, they are interleaved with ester bonds providing non-proteinaceous molecular architecture less recognizable by the immune system, that means low or zero immunogenicity of the polymers. 

#### 3.1.4. Pseudoproteins

Pseudoproteins (PPs) represent a family of the artificial AA-based polymers which have “head-to-head”-“tail-to-tail” orientations of AAs within the backbones. Such type of orientation of AAs provides non-proteinaceous architecture of artificial AABPs also less recognizable by the immune system [75,76,77,78].

**Figure 10:**
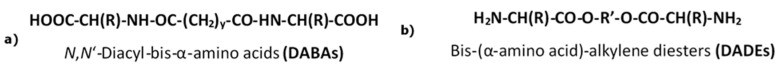
Dimerized forms of AAs with (**a**) “head-to-head” and (**b**) “tail-to-tail” orientations of AAs.

The said orientations of AAs in the macromolecules can be achieved using two types of dimerized AAs (Figure 10) as basic building blocks—either (a) diacyl-*bis*-α-amino acids or (b) *bis*-(α-amino acid)-alkylene diesters [75]:

The orientation of AA is considered assuming that α-amino group is a “head” and α-carboxyl group is a “tail”. Regardless the type of the dimerized monomer (Figure 10), the orientation of AAs after the construction of the polymeric backbones on their basis will be the same—the combination of “head-to-head” and “tail-to-tail” orientations.

**Figure 11:**
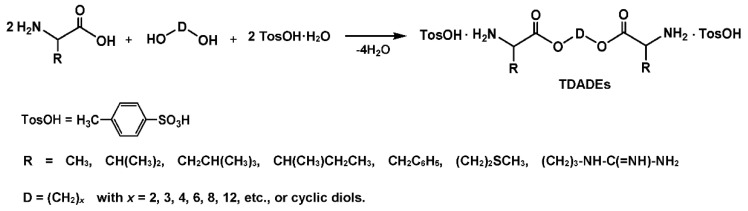
Synthesis of the TDADE monomers by direct thermal condensation of AAs with fatty diols in the presence of *p*-toluenesulfonic acid.

Among two types of the monomers given in Figure 10, much more universal from synthetic and technological points of view are *bis*-(α-amino acid)-alkylene diesters (diamine-diester, DADEs). The latter are synthesized by direct thermal condensation of two moles of α-amino acids with one mole of diols in refluxed organic solvent like benzene, toluene, or cyclohaxane in the presence of two moles of *p*-toluenesulfonic acid (Figure 11). After this reaction the DADEs are obtained with excellent yield (90–95%) as stable di-*p*-toluenesulfonic acid salts (TDADEs) [75,76,77].

The TDADEs are key bis-nucleophilic monomer for synthesizing a virtually unlimited number of PPs—representatives of a huge family of ester-polymers known as having reasonable biodegradation rates through the hydrolysis of ester moieties [75,76,77].

**Figure 12:**
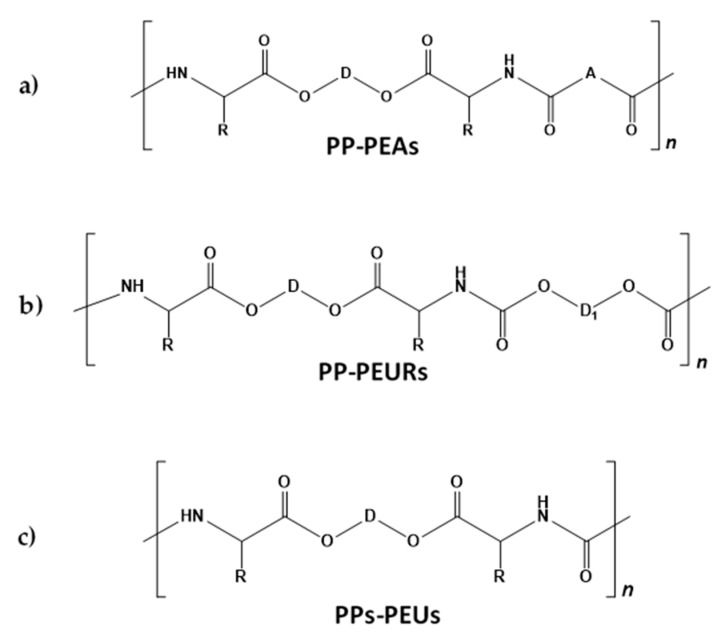
The structures of pseudoprotein (PP)–poly(ester amide)s (PEAs) (**a**), PPs–poly(ester urethane)s (PEURs) (**b**), and PPs–poly(ester urea)s (PEUs) (**c**).

After interacting the TDADEs with various bis-electrophiles, three basic classes of the PPs such as poly(ester amide)s (PEAs), poly(ester urethane)s (PEURs), and poly(ester urea)s (PEUs) (structures in Figure 12), as well as new AABB type PDPs [75,76] were obtained. Two techniques of the AABB type SGP such as interfacial polycondensation (IP) and solution active polycondensation (SAP) were employed for synthesizing the PPs [75,76,77,78].

More details of the synthesis, properties, and applications of the PPs including functional PPs are presented in [75,76,77,78]. For example, PPs have been employed as “artificial skins” and used as a wound bandage to treat poorly healing wounds. Elastomeric PP derivatives showed excellent blood and tissue compatibility, being in some cases employed as coatings of vascular stents and considered for cardiovascular applications. PPs have also been considered as barriers for preventing undesirable adhesions after surgery. PPs have some advantages over biodegradable polyesters and therefore appear as highly promising materials for the fabrication of resorbable bioengineering scaffolds. The specific PP-PEUs family may show outstanding mechanical properties since degradable polymeric constructs with moduli in the range of 6.0 GPa have been described. Therefore, these new materials can provide structural support and facilitate new tissue regeneration strategies in load bearing applications.

### 3.2. Biomimetics of Nucleic Acids—Polyphosphoesters

Polyphosphoesters are cost-effective, multifunctional, and environmentally friendly artificial polymers made of phosphoric acid. They are important materials for the applications in industry, agriculture, and medicine. Various polyphosphoesters such as poly(alkylene H-phosphonate)s, poly(alkylene phosphate)s, poly(alkyl or aryl phosphonate)s, and poly(alkyl phosphite)s and poly(alkyl phosphinite)s were designed during last 3–4 decades [96]. 

Polyphosphoesters are important polymers for applications as biomaterials since similar to naturally occurring biopolymers (e.g., nucleic acids) they show excellent biocompatibility. The polyphosphoester backbones similar to polyesters degrade through nonspecific or specific (an enzyme catalyzed) hydrolysis and are considered as biodegradable [97,98]. Biodegradable polymers are of a high interest for the applications in medicine and as environmentally friendly materials. Poly(phosphate ester)s as biodegradable polymers were reported in late 80s for constructing drug-delivery devices [99]. 

Poly(alkylene phosphate)s having backbones depicted below

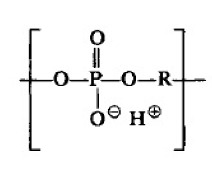

are basic parts of two important groups of biopolymers: nucleic acids and teichoic acids. For synthesizing the poly(alkylene phosphate)s initially (poly(alkylene phosphonate)s) are synthesized which are then transformed into the corresponding phosphates.

**Figure 13:**
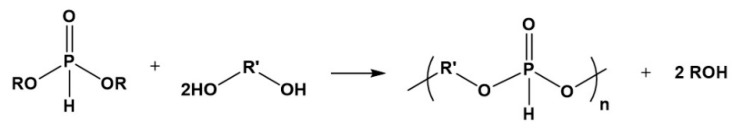
Synthesis of polyphosphoesters by polycondensation of dialkyl phosphonates with diols.

**Figure 14:**
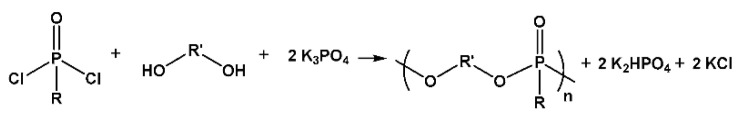
Synthesis of polyphosphoesters by polycondensation of substituted phosphonic dichloride with diols.

Polyphosphoesters such as poly(alkylene H-phosphonate)s) could be obtained by polycondensation of dialkyl phosphonates with diols according to Figure 13 [100,101,102,103]:

Polyphosphoesters could also be synthesized by polycondensation of substituted phosphonic dichloride with aromatic diols (bis-phenols) (Figure 14) [104].

When using tyrosine-based dipeptides depicted in Figure 9 above, bis-phenols biodegradable polyphosphates completely composed of physiological building blocks were synthesized [105]. 

**Figure 15:**
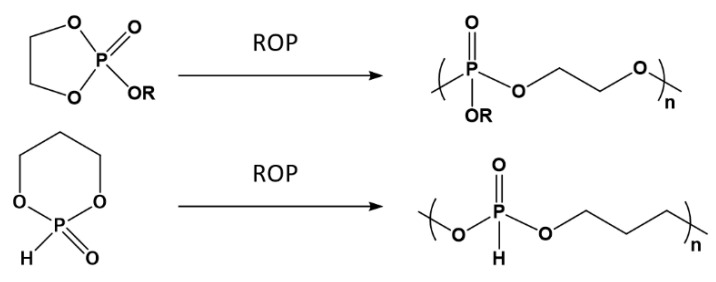
Synthesis of polyphosphoesters by ROP.

Polyphosphoesters can be obtained also by ring-opening polymerization (ROP) of cyclic phosphoranes [97,101] as depicted in Figure 15. 

**Figure 16:**
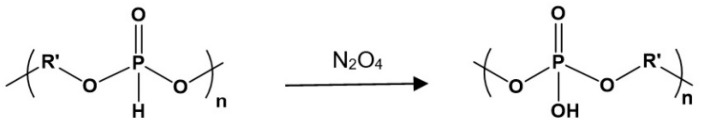
Converting the of polyphosphites into polyphosphates by oxidation with N_2_O_4_.

Polyphosphites were further transformed into corresponding polyphosphates by oxidation with N_2_O_4_ [100] (Figure 16).

**Figure 17:**
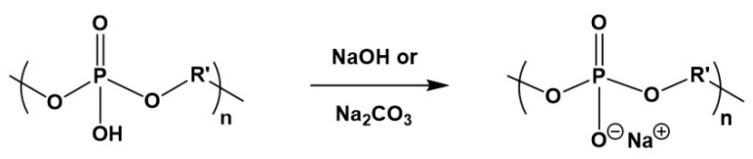
Transformation of polyphosphate-polyacids into corresponding salt by neutralization with NaOH or Na_2_CO_3_.

Polyphosphites were also transformed into polyphosphates by their chlorination with gaseous Cl_2_ with subsequent transformation of the P-Cl polymers into intermediate P-imidazole derivatives by the interaction with an excess of imidazole; the imidazole derivatives were then hydrolyzed and converted into free polyacids (P-OH derivative) [103]. The free polyphosphate-polyacids could be converted into to their sodium salts (Figure 17).

**Figure 18:**
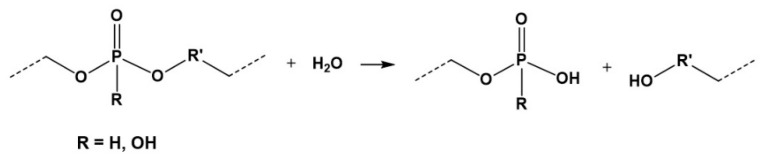
Hydrolytic degradation of polyphosphoesters.

The polyphosphites and polyphosphates have a potential for biomedical applications as biodegradable materials taking into account the hydrolytic mechanism of their cleavage (Figure 18) [96,98]. 

It is important that after hydrolysis polyphosphoesters release nontoxic products such as derivatives of phosphoric acid and diols [96,98] or physiological debris such as derivatives of AA tyrosine [105]. This, along with a low immunogenicity of artificial (synthetic) degradable polymers [75,76,77], make polyphosphoesters highly promising materials for biomedical applications, mostly as drug carriers apt for intracellular delivery of drugs and other bioactive molecules [96,97]. 

Amphiphilic biodegradable biomaterials polyphosphate graft copolymers having tunable quantity of cholesteryl groups to control hydrophobicity of polymeric chains were designed for solubilizing hydrophobic drugs such as e.g., anticancer drug paclitaxel. In aqueous solution the polymers formed stable nanoparticles containing paclitaxel. The graft polymers were examined with some cell cultures and showed no cytotoxicity. The graft polyphosphate copolymers are important for the biomedical and pharmaceutical fields as effective drug delivery systems [106].

## 4. Hybrid Fabrication and Emerging Systems

Selection of the fabrication technique should consider the optimal architecture of the scaffold which depends on the final application. Porous scaffolds with an interconnected pore structure are ideal to guarantee the cell in-growth and the flow of nutrients, and are basic for peripheral nerve, blood vessels, bone regrowth, and ECM deposition. Porosity should be around 90% [107], while pore size may be varied between 200 and 400 μm for bone tissue [108], 50–200 μm for smooth muscle [109], and <50 μm for fibrous tissues [110]. Nanofibrous scaffolds appear ideal for skin, cartilage, ligaments, muscles, and veins [111]. These can mimic the ECM, facilitate cell attachment due to their high surface-to-volume ratio and the high aspect ratio of constitutive nanofibers, and provide a guiding alignment (as desirable for example for neurite growth). Usually, mixed nanofibers are employed in order to enhance properties of individual scaffolds. Nevertheless, small pore size and limited thickness are some specific limitations of the nanofibrous scaffolds. 3D geometrically complex scaffolds with high precision of spatial parameters can also be obtained by successive printing of 2D layers. Perfection of the solid free-form scaffold would depend on the selected 3D printing technology and the resolution of the device for image acquisition.

Probably, solution electrospinning is one of the most powerful processing technologies to render suitable materials for tissue regeneration. Basically, the process takes advantage of its great versatility and low cost. The applicability in tissue engineering was demonstrated when collagen fibrils were mimicked [28] by a simple stretching under a high electric field of a pertinent polymer solution. Nevertheless, electrospinning has some drawbacks that limited its exclusive use, between them it should be indicated the usual low porous and compact structures that are obtained. The versatility of electrospinning allows using different approaches to overcome some difficulties. As interesting complex methodologies can be mentioned: (a) the combination of electrospinning and electrospraying to deposit cells inside the polymer matrix and overcome the problem of cell penetration [112], (b) the combination of solution and melt electrospinning [113] or even melt electrowriting [114] to render a porous and bulky scaffold, and (c) the incorporation of a sacrifice polymer, such as water soluble polyethylene glycol, to increase porosity [5]. This incorporation can be performed by means of co-electrospinning using either two opposite spinnerets with a common rotating collector [115] or a coaxial electrospinning [116]. The combination of typical melt extrusion and solution electrospinning has also been applied to guarantee porosity through the presence of the more voluminous extruded microfibers [117]. In fact, the integration of electrospinning with other technologies allows to get multifunctional 3D systems having nano and macro-features and a clear improvement of biological performance [118].

### 4.1. Solution Based Fabrication and Electrospinning

Different techniques use solvents for the preparation of porous scaffolds, between them are relevant the thermally induced phase separation process (TIPS), the solvent casting, and particulate leaching method and the solution electrospinning technique. This attracts more attention due to being an easy tuning process with a high capacity to work with a wide range of materials (i.e., these can be properly selected according to the properties of the tissues that should be regenerated) [7,119]. All the mentioned techniques bring advantages and disadvantages concerning material processing. For example, TIPS require low boiling point solvents, temperature being a crucial parameter to provide porous templates for nerve, muscle, tendon, ligament, intestine, bone, and teeth regeneration [120]. TIPS is based on the thermodynamic demixing of a homogeneous polymer solution into polymer-rich and polymer-poor phases, the solvent in the last phase being subsequently eliminated by extraction, evaporation, or sublimation to leave behind a highly porous polymer network. Porosity and pore morphology can moderately be controlled (e.g., pore size varied between 10 and 2000 µm), and consequently some applications (especially drug release and cell growth) become limited [121]. Similar to this technique, phase separation induced by a nonsolvent has also been applied for scaffolds based on biodegradable aliphatic polyesters [122]. The desired scaffold architecture can be obtained through the addition of various porogenic agents, which should be easily removed by leaching once the initial polymer solution has been solidified. Pore size and interconnectivity of the derived materials are easily controlled by the geometry and concentration of the added porogenic agents. However, this simple processing technique has limitations concerning the biopolymer systems, the lack of an accurate control of microstructure (e.g., limited pore size) and the presence of residual solvent [123]. 

Electrospinning attracts great attention for fabrication of micro/nano fibrous materials similar to human tissues [124,125] due to its simplicity and the good characteristics of the derived scaffolds (e.g., high porosity, high surface area to volume ratio). Although within this technique it is possible to provide bone, skin, nerve, and cardiac tissues, it should be indicated as a major challenge the difficulty to find a suitable solvent to sufficiently evaporate and stabilize the spinning process [126]. However, electrospinning of natural polymers like collagen, chitosan, and gelatine make this technique to surpass others to accurately mimic natural ECMs [28]. Physical and mechanical properties of fibrous electrospun structures can easily be modified by incorporating another polymer to develop hybrid blends or composite systems. As an example, the addition of PLA improves the processability and mechanical properties of collagen for vascular prosthesis applications [127]. 

**Figure 19:**
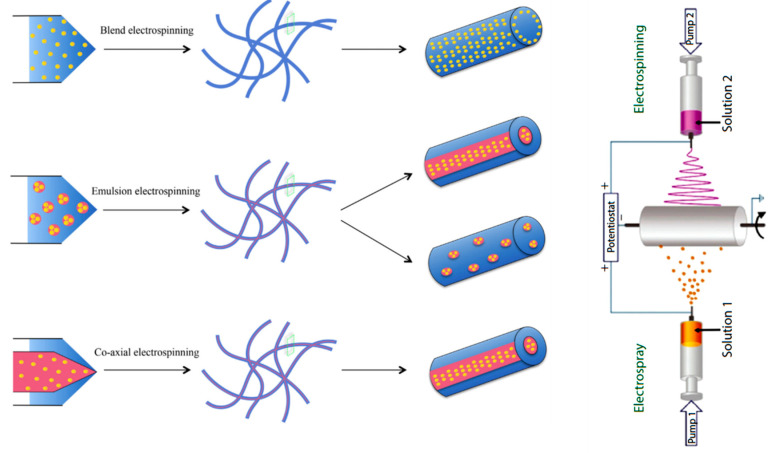
Schematic illustration of different electrospinning techniques used for fabrication of hybrid scaffolds. Reprinted with permission from [130,132].

Besides properties and viscoelasticity of the selected polymer, which play an important role in the electrospinning process, selection of solvent, applied voltage, flow rate, spinneret-collector distance, and collector geometry are essential to get scaffolds with the desired properties to accomplish with the final application [124]. For example, the design of a scaffold for the medial layer of a native artery needs the use of aligned nanofibers. In this case, smooth muscle cells and extracellular matrix fibrils are cylindrically aligned by means of a rotatory collector. The final geometry favors vasoconstriction and vasodilation in response to the corresponding stimuli [128]. It is clear that the size scale, porosity, and orientation of scaffolds can be modified to influence cell functions such as adhesion, proliferation, and migration. Nevertheless, even a greater enhancement over the control of cellular function can be achieved by attaching bioactive molecules to the surface of the scaffolds. Different electrospinning techniques including emulsion electrospinning, coaxial electrospinning, blow assisted electrospinning, and combination of electrospraying/electrospinning [2,129,130] have been proposed to extend the functions of electrospun fibers and provide new hybrid systems as schematically shown in Figure 19 (left images). It is interesting the capacity to modify the surface of electrospun fibers by adding nanoparticles or functional small molecules by means of electrospraying [131,132] (Figure 19 (right image)) [131], which uses low solute concentrations to avoid chain entanglements and favor the formation of droplets. 

Addition of another polymer or inorganic components such as HAp nanocrystals, bioglass, silica, and calcium carbonate are considered as new potential ways for generating nanofibers with favorable osteoblastic adhesion and cell growth besides the high water affinity and good mechanical performance [124]. Recently Cui et al. [133] used the electrospinning technique to provide an artificial periosteum for accelerating the bone regeneration by means of a hybrid hydrogel. When a bone defect takes place, the periosteum performance is affected and nutrition to support bone formation needs to be improved. In this case the hybrid system based on calcium phosphate nanoparticles as an inorganic component and gelatine-methacryloyl as an organic component were prepared using the electrospinning technique. The final hydrogel was obtained by immersion of the fibrous mat into the photo crosslinking solution and subsequent exposure to UV light. Mechanical properties, ion release, and mineralization on the skin with simulated body fluid demonstrated that this biomimetic organic-inorganic hybrid hydrogel had good capabilities to promote osteogenic differentiation and angiogenesis. 

**Figure 20:**
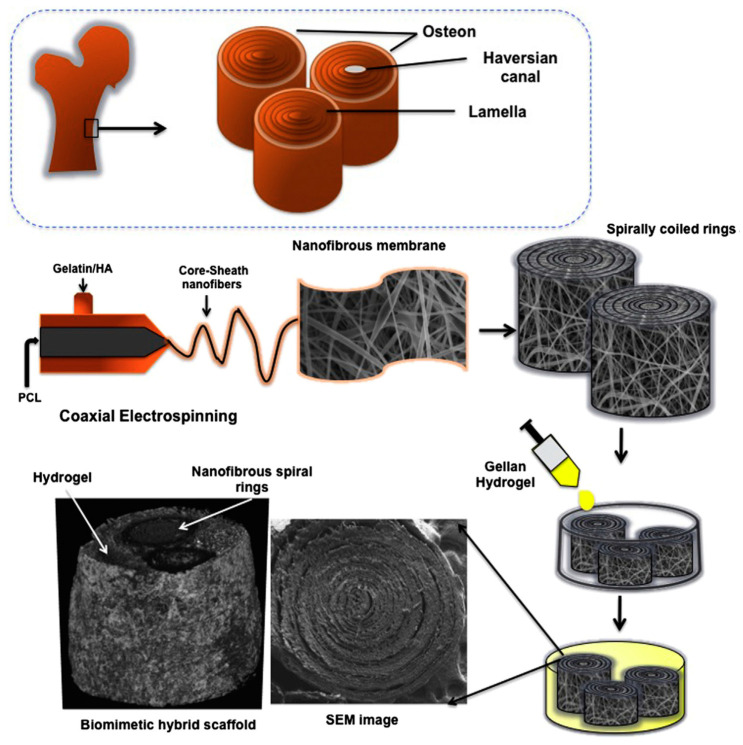
Microstructure of bone extracellular matrix (ECM) and schemes showing the two main processing steps to get biomimetic three-dimensional hybrid scaffold. Reprinted with permission from [134].

3D core sheath structures of PCL nano- and microfibers with gelatine and HAp nanocrystals were fabricated to mimic the nano-hierarchy of bone [134]. For preparation of this 3D structure, first, 10 wt-% gelatine and 7 wt-% PCL solution were prepared in trifluroethanol and then the coaxial electrospinning was employed to fabricate the fibrous membrane and spirally coiled ring. In addition, the HAp was added to the gellan and the hydrogel of gellan/HA was prepared using lyophilization at −80 °C for 24 h. PCL is able here to enhance the mechanical properties of gelatin nanofibers, whereas HAp improves the capability to promote mineralization. The characteristic osteon morphology of cortical bone was successfully mimicked by using the coaxial nanofibrous membranes to produce spirally coiled rings, which were finally covered by a gellan hydrogel. Scaffolds with an appropriate 3D-bone architecture for osteocytes and good mechanical properties for bone tissue regeneration were therefore provided. Different steps involved in the fabrication of this complex scaffold are illustrated in Figure 20. 

Another interesting approach is the electrospinning of a polymer (e.g., PCL) to obtain a nanofiber deposition on the surface of 3D printed layers (e.g., PLA). This gives rise to a mechanically supported system that may be, for example, interesting for musculoskeletal tissues [135]. The derived hybrid scaffold had an enhanced cell attachment rate and provided a microtopological cue for cells to organize into a patterned structure.

As discussed above, PCL and PLA fibers extensively showed the reinforcement of natural biopolymers and improved the cell adhesion and even proliferation processes. Polyamide (PA) may be also a suitable candidate in this area due to its easy processability, biocompatibility, and superior mechanical properties [136]. Hybrid nanofibrous scaffolds of poly(hexamethylene adipamide) (PA66) and chitosan have been prepared by solution electrospinning and showed a biomimetic nature with high cell viability and high regeneration of pre-osteoblast cells. Furthermore, this nonuniform hybrid structure (PA66 and chitosan fibers with diameters around ≈200 and ≈40 nm, respectively) showed good mechanical performance and water wettability, which are crucial requirements for the development of biomimetic scaffolds. 

### 4.2. Melt Electrospinning and Combined Methods

Melt electrospinning overcomes the disadvantages of solution electrospinning since the use of potential toxic solvents can be avoided. Among the advantages of this technique, there are still limited interest for biomedical applications like bone regeneration and tissue engineering due to the large fiber diameter of the electrospun fibers and the necessity to work at high temperature (i.e., obviously above the melting point of polymer) [137,138]. Process conditions in melt electrospinning mainly depend on the polymeric system and the selected configuration. The main parameters include processing temperature, spinneret-collector distance, applied voltage, flow rate, and spinneret diameter in the simplest melt electrospinning process [138]. 

**Figure 21:**
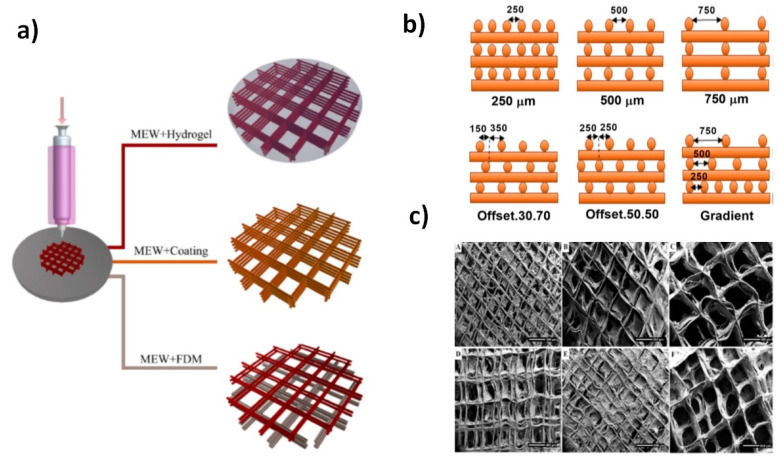
Scheme showing the preparation of scaffolds with different hybrid manufacturing methods based on melt electrowriting (MEW) (**a**) and pattern structures with different offset and pore sizes (**b**). Examples of derived architectures combining MEW and coating of calcium phosphate are also shown in (**c**). Reprinted with permission from [140,142].

Different melting configurations have been developed since fusion is fundamental to get an initial jet. Therefore, possibilities include the use of a ceramic/electrical heater, an oil circulating heating system, a glass syringe dotted with a heat gun, and gas assisted and CO_2_ laser heating system [138]. Control of polymer degradation and viscosity during melt processing are crucial for choosing the most suitable configuration. In addition to the heating system, different configurations of the collector can provide the desired products and morphology. As an example, the use of the gap method of alignment for collection can lower the PCL fiber diameter from 2 µm to 270 nm [139]. Among the different configuration and techniques for obtaining micro and nanofibers, melt electrowriting was also developed for providing the designed pattern. This technique is similar to a 3D-printing and takes also advantage of a computer-aided design (CAD) [140]. This allows a correct and continuous alignment of the nozzle and collector for the deposition of the extruded filament. The layer by layer deposition is repeated until the desired 3D object is fabricated. The increase of the applied voltage allows further reduction in the filament diameter, which is a crucial point to improve the resolution of the melt electrowriting process. New opportunities for fabrication of different biomimetic tissues are possible through hybrid manufacturing based on melt electrowriting and the incorporation of hydrogels, coatings, or its combination with fused deposition modelling (FDM) (Figure 21a). In FDM technology, the melt extrusion method is used to deposit filaments of thermoplastics according to a specific pattern. Different architectures are fabricated with customization of CAD with different porosity and physical/mechanical properties (Figure 21b). In addition, combining this technique with the simple coating resulted in composite of fiber with designed structure which render a suitable scaffold with both cell proliferation and mechanical performances (Figure 21c).

Novel scaffolds can be designed with tailored properties and particular features by combining different techniques since in this way it is possible to take profit of the advantages provided by each of them. For example, co-electrospinning was applied to prepare a PLA_PEG/PLA_silk fibroin scaffold [141]. Specifically, PLA was melt electrospun with PEG as plasticizer while solution electrospinning of PLA_silk fibroin was also performed to develop a scaffold consisting of micro- and nanofibers. This approach makes feasible a great control on the morphology and geometry of deposited fibers.

Efforts have also been addressed to improve the mechanical performance of hydrogels because they should play a relevant role in tissue regeneration due to their similarities to human tissues and the easy encapsulation of cells in their hydrated structures. The use of solvent free processes appears as a significant tool to enhance their properties as mechanical support and capacity to mimic the function of fibrous tissues. To this end, hybrid scaffolds have been fabricated by combining the preparation of fibers using melt electrowriting and the infiltration of the hydrogel in the manufactured fibers [142].

Melt electrowriting has also been combined with solution electrospinning to get biomimetic structures showing both nano- and microfeatures and to provide the sufficient mechanical performance [143]. One of the most attractive biodegradable polymers employed in melt electrospinning is PCL since it has a low melting temperature. Different examples concern its use as mechanical support of collagen. For example, PCL/collagen nanofibers were prepared by solution electrospinning and then PCL layers were deposited above by melt electrowriting. Nandakumar et al. have recently reported another interesting combined process [144] to get scaffolds with a great potential for bone tissue engineering. In this case, melt electrowriting was employed to prepare a mechanically stable structure, solution electrospinning was used to provide a random mesh mimicking ECM, and a biomimetic coating was finally applied to increase bioactivity of the polymer used (Figure 21c). 

Melt electrospinning of PCL and solution electrospinning of silk fibroin have also been combined to fabricate the nano/microfibrous scaffolds with good mechanical properties and applicable in bone regeneration [145]. Small pores formed by silk fibroin nanofibers in these composite scaffolds have a positive effect on the cell growth, providing a suitable environment for cell proliferation, adhesion, and in vitro differentiation into osteoblasts. 

**Figure 22:**
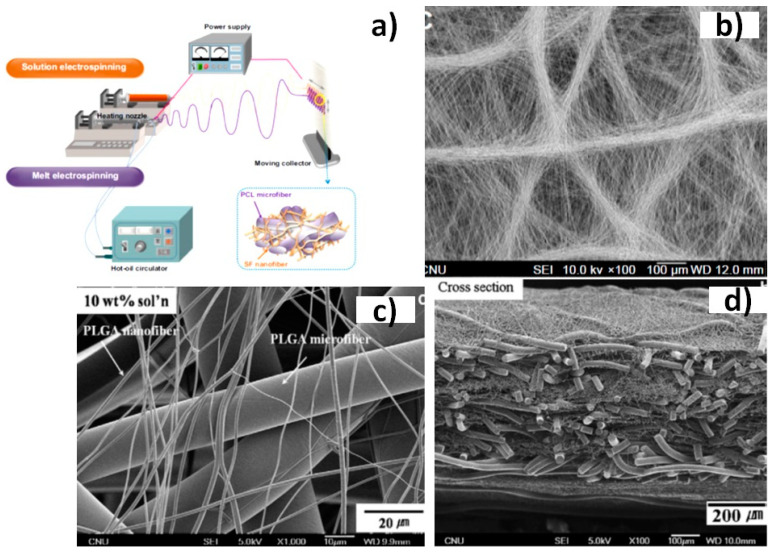
Schematic diagram of the hybrid solution-melt electrospinning process (**a**) and different fabricated hybrid scaffolds of silk fibroin/poly(ε-caprolactone) (PCL) (**b**) and poly(lactic-*co*-glycolic acid) PLGA (**c**) and (**d**) nano/microfibrous composites. Reprinted with permission from [113,145].

Sandwiched or interpenetrated hybrid structures have also been designed to improve mechanical properties of scaffolds. These constructs can also be obtained by combining solution and melt electrospinning (Figure 22a). Composite scaffolds having micro- and nanofibers of poly(lactic-*co*-glycolic acid) (PLGA) (Figure 22b–d) showed a high mechanical performance as consequence of the strong unions existing between the entangled fibers in the sandwich structure [113]. 

## 5. Specific Applications in Tissue Regeneration

A wide range of applications can be covered by biodegradable scaffolds based on synthetic and natural polymers: healing of skin and wounds, reconstruction of bone, cartilage, and muscles, reparation of the nervous system, regeneration of vasculature, and even drug delivery and gene therapy. Regeneration of tissues and organs can be achieved through stem cell-based therapies, which still have different challenges such as cell death and uncontrolled differentiation. Other challenges that must be solved concern uncontrolled cell differentiation, severe death of cells, and even a poor yield on the functional engraftment. Nevertheless, the potential of new therapies to regenerate different kinds of tissues and organs is enormous and effective clinical translation seems imminent.

Scaffolds based on advanced functional biomaterials are nowadays designed to deliver stem cells to targeted tissues/organs. In this way, cell survival and differentiation can be enhanced as well as the integration to the host tissue. All these features demonstrated the great clinical potential of regenerative therapies based on the use of stem cells. Wang et al. [146] have recently summarized the main sources of stem cells for transplantation, presented the current state of the art in biomaterial design for stem cell delivery, and provided critical analysis for existing functional biomaterial scaffolds. Applications to the cardiovascular, neural, and musculoskeletal systems have also been highlighted with recent nonclinical studies and clinical trials.

Bone tissue regeneration tries to mimic the intramembranous ossification by means of 3D scaffolds and the osteogenic differentiation of seeded mesenchymal stem cells (MSCs) [147]. Nevertheless, different problems must still be solved, these being mainly related to the lack of functional vascular supply upon implantation and even the sealing of the pores of the scaffold [148]. Interesting new approaches are based on the so named “development engineering” [149]. In this case, MSCs followed a chondrogenic differentiation and formed a cartilaginous template, which at the end was remodeled into bone. The advantage of the new approach is the fact that chondrocytes can function in environments with an oxygen deficit (i.e., avascular tissues [150]) as those that can be found after the scaffold implantation. Sheehy et al. [148] have recently reviewed the biomaterials, cells, and signaling factors that can be used to develop cartilaginous grafts able to promote endochondral bone formation as well as the manufacturing of anatomically shaped templates, the fabrication of mechanically reinforced constructs, and the production of gene-activated scaffolds to accelerate bone regeneration.

Healing of tendons is rather problematic due to its low cellular and hypo-vascular nature that leads to a disorganized ECM and tissues with low biomechanical properties. New tendinopathy therapeutic strategies are focused to design appropriate biomimetic scaffolds and choose the suitable cell source. Mesenchymal stem cells and fibroblasts are widely employed in tendon tissue engineering despite that in vivo transplantation of stem cells shows tendon ossification problems and fibroblasts have a limited proliferative capability. Nevertheless, other cells are investigated with highly promising results being found by using amniotic epithelial stem cells (AECs). These displayed a high differentiation capability, especially for tenogenic tissues [151]. 

Recently, it has been demonstrated that polymeric scaffolds based on PCL, PLA, or PLGA were able to support AECs colonization, distribution, and proliferation [152]. Electrospinning using a high-speed rotating collector allowed to get highly aligned fibers which were demonstrated to be beneficial in terms of tenogenic differentiation [153]. Namely, interactions between AECs and biocompatible aligned fibers can lead to suitable bio-hybrid constructs for tendon tissue engineering.

Trachea is a fundamental cartilaginous construct for living beings that causes respiratory problems when damaged and causes cancer if disorders are not cured in time. Tracheal tissue engineering features and outcomes have recently been overviewed by Dashmana et al. [154]. Human epithelial cells cocultured with articular chondrocytes within fibrin scaffolds have been proven to be highly efficient [155]. The epithelial cell tissue is basic since it acts as a barrier and regulates also the metabolic function of airways, while chondrocytes enhance the biomechanical strength of the scaffold. Optimal characteristics of scaffolds are a porosity close to 90%, a pore size between 5 and 100 μm, a high pore connectivity, good mechanical strength (212 ± 18 N), a high flexibility (tensile modulus around 11 MPa), and obviously biocompatibility, biodegradability, and non-immunogenicity [156]. First polymers employed as porous matrices were based on PGA [157]. Subsequently, systems based on its modification with other polymers such as alginate [158] were developed as well as scaffolds based on collagen and gelatin [159], silk [160], fibrin [161], or PCL [162].

**Figure 23:**
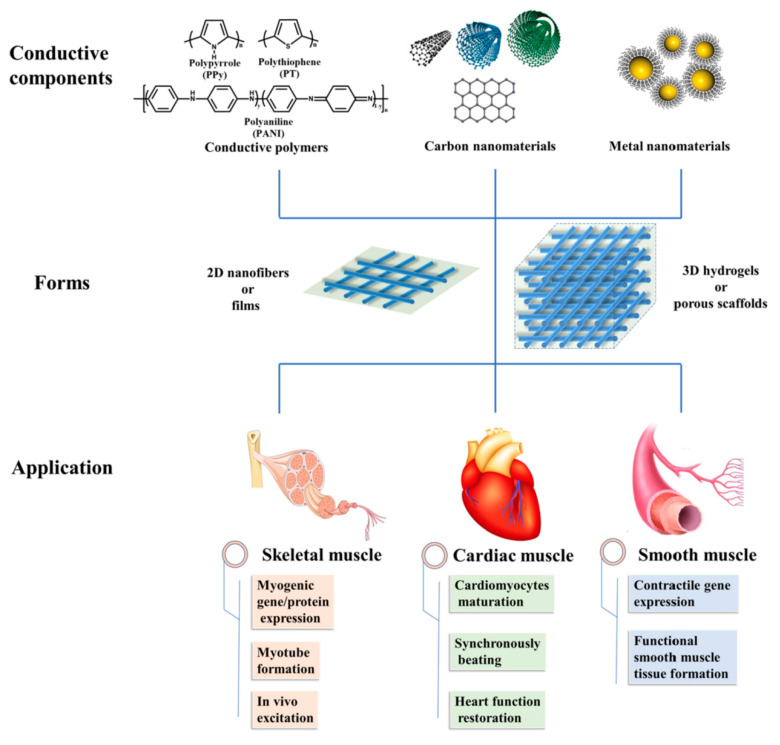
Main conductive materials, scaffold forms, and applications in muscle tissue regeneration. Reprinted with permission from [113,145].

Muscle tissue injuries mainly affect force generation, body movements, and even organ functions. Development of scaffolds having conductive polymers are nowadays one of the more promising strategies for muscle tissue engineering since conductivity is a key factor to enhance muscle tissue formation [163] (Figure 23). Cells can acquire a specific charge with the use of conductive materials. Under local electrical fields ion transfer and movement across cell membranes may be promoted, favoring cell attachment and proliferation as well as protein expression [164].

Spinal cord injury (SCI) is nowadays one of the major health problems that is receiving attention in the neuroscience field. Cell transplantation supported by biomaterial scaffolds provides a mild microenvironment that becomes a promising therapy for nerve tissue repair [165]. Probably, MSCs are the more common transplanted cells [166] but their differentiation into nerve cells is problematic and in addition they can hardly survive in the environment of injured tissue [167]. Therefore, interesting attempts have been carried out using neural stem cells [168]. Scaffolds were prepared by mixing collagen and silk fibroin and the subsequent cross-linking. In this way advantages of collagen (i.e., good biocompatibility and degradation rates without provoking immunogenicity) and fibroin (i.e., toughness and flexibility) could be preserved.

**Figure 24:**
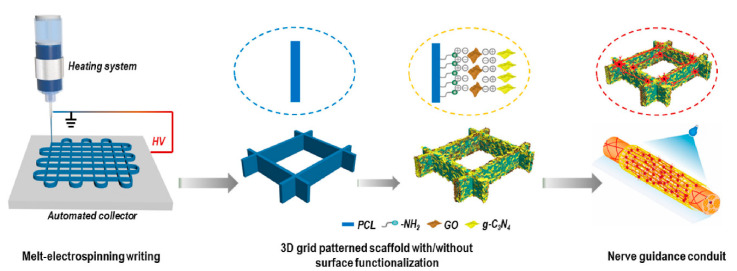
3D grid patterned PCL scaffolds decorated with visible-light photocatalyst as nerve guidance conduits (NGCs) for promoting peripheral neural regeneration. Reprinted with permission from [113,145].

Development of nerve guidance conduits (NGCs) is currently involving great efforts to make feasible peripheral neural regeneration. Appropriate design should consider important aspects like anisotropy, photocatalytic stimulation, and self-assembly. An interesting work is described by Cheng et al. [169] (Figure 24), being in this case electrowriting employed to get anisotropic, microfibrous, PCL architectures. Photocatalytic stimuli were incorporated by post-decoration with graphitic carbon nitride. The derived patterned structure was able to accelerate and lead to neurite outgrowth.

Biomimetic vascular structures are currently being developed to solve one of the major human health problems. These structures should accomplish strict requirements about structural stability and continuity to keep a correct blood flow [170]. 3D bioprinting technology appears as a promising fabrication method due to its great printing flexibility and low cost. Engineered blood vessels have for example been prepared by using a hybrid bioink based on gelatin, sodium alginate, and carbon nanotubes. Cylindrical scaffolds with inoculated fibroblast in both inner and outer walls were manufactured by combination of a vertical directional extrusion nozzle and an axial rotation of the motor module [39].

Emerging roles of gene-editing and nanotechnology systems in vascular tissue engineering have recently been reviewed by Huang et al. [171]. Discussion is extended to the improvements focused to solve the existing gaps in stem cell and regenerative therapies, and also to the current 3D printing advances for manufacturing engineered tissues.

A meniscus injury can lead to cartilage degeneration and ultimately to osteoarthritis [172]. Deficient blood supply difficult the healing process, a surgical intervention often being necessary. Commercial implants have different drawbacks such as the incapacity to restore completely and lead to a permanent replacement of the natural tissue. Fortunately, recent advances in tissue engineering open great perspectives for meniscus repair [173]. Alginate gel scaffolds [174] and silk scaffolds [175] have been evaluated due to their biocompatibility but biomechanics should be improved. A tailored scaffold produced by 3D printing and based on silk fibroin, PCL, and synovium-derived mesenchymal stem cell has recently been developed, with good autologous MSC recruitment, differentiation, regeneration of meniscus, and chodroprotection being found [176].

Cutaneous tissue protects the body against infectious agents while minimizes the evaporation of biofluids. The skin is usually able to self-heal due to the presence of basal cells between dermis and epidermis. These enhance the replacement of injured cells with functional keratinocytes but have a limited reparative action when extensive diseases occur (e.g., burns, diabetes, traumas, etc.) [177,178]. Different efficient scaffolds have been proposed for cutaneous tissue engineering, being in general proposed hybrid systems based on natural and synthetic polymers to fulfil all biological activities [179]. For example, electrospun scaffolds based on natural eggshell membrane (ESM), PCL, silk fibroin, and *Aloe vera* showed appropriate physicochemical properties and biological activity to be employed as a biomimetic scaffold for the regeneration of tissue [180].

Connective tissue of human vocal fold can be easily damaged leading to hoarseness and the alteration of vocal biomechanics. Ideal materials for biomimetic scaffolds are in this case highly limited due to the difficulty to get an appropriate stiffness [181]. Typical PLA and PCL materials have a too high elastic modulus compared with that shown by vocal folds (i.e., 50–730 kPa). Therefore elastomeric biomaterials are considered, being interesting poly(glycerol sebacate) due to its promising results for soft tissue applications [182], and even more its blend with thermoplastic polyurethanes that facilitated the electrospinning process and slowed the final degradation rate [183].

## 6. Conclusions

Accurate selection of materials is essential to develop appropriate scaffolds for tissue engineering applications. These materials should allow incorporating growth factors and host cells while providing cues to guide cell adhesion and proliferation. Natural polymers like fibrin, keratin, chitosan, and the different forms of collagen have been successfully employed. Probably, hydrogel scaffolds appear the most ideal matrices to culture cells and produce in vitro tissues, polysaccharides being highly appropriate due to their hydrophilicity, biodegradability, and biocompatibility. Bio-responsive functions and similar properties to ECM can be attained although chemical modifications are still necessary to facilitate the cell attachment. 

Despite their higher cost, the development of peptide hydrogels is gaining attention. Efforts are nowadays also focused on the incorporation of electroactive polymers and conductive nanoparticles due to the beneficial effect of electrostimulation on tissue regeneration. The use of elastomeric materials has also a high interest for tissue engineering applications as a consequence of their chemical inertness and their suitable mechanical properties.

Among the artificial degradable polymers designed for biomedical applications one of the most promising are polymers mimicking naturally occurring polymers—proteins and nucleic acids. These biomimetic systems have numerous advantages over the natural polymers such as wider range of material properties, higher safety and higher reproducibility, excellent compatibility with tissues, lower price, to name a few. This makes the biomimetics highly promising for wide biomedical applications as resorbable surgical materials and drug delivery systems.

Considering the design of polymer for desired biomimetic applications, the selection of processing and fabrication methods is primordial. Among different techniques, electrospinning and more specifically eco-friendly melt electrospinning and writing attract more attention to provide a nontoxic environment for biomimetic applications. Further improvements are possible combining two or more techniques, rendering products with suitable physicochemical, cell proliferation, and mechanical properties. For example, electrospinning of hybrid systems, coelectrospinning and coaxial electrospinning, two step hydrogel production, combination of melt electrowriting and electrospinning have been demonstrated as effective ways for fabricating new and modified scaffolds for tissue engineering.

Synthetic and natural polymers are being continuously tested and modified to design biomimetic scaffolds for a wide range of tissue engineering applications such as healing of skin and wounds, reconstruction of bone, cartilages and muscles, reparation of the nervous system, regeneration of vasculature, and even drug delivery and gene therapy. Recent designs are focused to create multifunctional structures able to provide complex biological functions. New therapies can potentially regenerate many types of tissues and organs, but some limitations like acute cell death, uncontrolled differentiation, and low functional engraftment yields still need attention. Efforts should also be focused to improve the understanding of interactions between cells and ECM, to tune the structure of scaffolds in order to simulate more accurately the ECM structures and topologies, and to insist on the preparation of stimuli responsive hybrid biomaterials.
